# Transposable Element *rbg* Induces the Differential Expression of *opaque-2* Mutant Gene in Two Maize *o2* NILs Derived from the Same Inbred Line

**DOI:** 10.1371/journal.pone.0085159

**Published:** 2014-01-09

**Authors:** Yan Chen, Zhiqiang Zhou, Gang Zhao, Xinhai Li, Liya Song, Na Yan, Jianfeng Weng, Zhuanfang Hao, Degui Zhang, Mingshun Li, Shihuang Zhang

**Affiliations:** 1 Department of Crop Genetics and Breeding, Institute of Crop Sciences, Chinese Academy of Agricultural Science, Beijing, China; 2 Beijing Key Lab of Plant Resource Research and Development, Beijing Technology and Business University, Beijing, China; Institute of Botany, Chinese Academy of Sciences, China

## Abstract

The recessive *opaque-2* mutant gene (*o2*) reduces α-zeins accumulation in maize endosperm, changes the amino acid composition of maize kernels, induces an opaque endosperm, and increases the lysine content of kernels. The quality protein maize (QPM) inbred line CA339 (*o2o2*) and an elite normal inbred line liao2345 (*O2O2*) were used to construct *o2* near-isogenic lines (NILs) by marker-assisted selection (MAS) using the co-dominant SSR marker phi057. Two specific *o2* NILs were constructed, named liao2345/*o2*-1 and liao2345/*o2*-2. However, the kernel phenotypes of the two *o2* NILs were different from each other. liao2345/*o2*-1 had the wild-type vitreous endosperm, which is similar to its recurrent parent liao2345, while the endosperm of liao2345/*o2*-2 was opaque, identical to typical *o2* mutant individuals. In comparison to their recurrent parent liao2345, the lysine concentration of liao2345/*o2*-1 was similar and the lysine concentration in liao2345/*o2*-2 was doubled. SDS-PAGE analysis indicated that liao2345/*o2*-1 had the same zeins ratio as liao2345, whereas the zeins concentration of liao2345/*o2*-2 was markedly lower. Sequence and transcript abundance analyses indicated that the CDS of two *o2* NILs are derived from CA339, but they have different promoters. The *O2* transcript of liao2345/*o2*-2 is largely inhibited because of an *rbg* transposable element inserted between the TATA box and initiator codon of liao2345/*o2*-2. We concluded that different crossing-over patterns during the process of *o2* NIL construction resulted in the different kernel phenotypes of the two *o2* NILs. We surmise that the reversion of liao2345/*o2*-1 to wild type was due to the recombination with the wild type liao2345 promoter during introgression and backcrossing. The *o2* mutant gene of donor (CA339) is a null mutant because of low *O2* expression. However, its CDS probably encodes a protein with normal function which can maintain the normal accumulation of zeins in maize endosperm.

## Introduction

Maize (*Zea mays* L.) is one of the most widely cultivated cereal crops in the world. Dependence on maize as a protein source puts people at risk of dietary protein deficiency because maize protein is deficient in two essential amino acids: lysine and tryptophan. Therefore, maize is a poor source of protein for humans and other monogastric animals [1]. In order to increase the quality of maize, a long-term selection experiment was initiated in 1896 by CG Hopkins at the University of Illinois [2] that resulted in the Illinois High Protein Strain (IHP). IHP has 2.5 times the amount of protein contained in normal maize and the majority of this increase is due to zeins, which contain no lysine [3]. In 1964, Mertz reported that the *o2* mutant gene can alter the amino acid composition in maize endosperm and double the lysine content [4].

Because the *o2* mutant gene can increase the lysine content in maize kernels, breeders began using this mutant to cultivate high-quality maize with more lysine. However this mutant maize had poor agronomic traits. This gene was cloned by transposon tagging [5], [6] and its sequence was published [7]. In the 1990s, the mechanism through which the *O2* gene regulates protein expression was intensively studied. The *O2* gene encodes a protein that has structural homologies to transcriptional activators [8]. The OPAQUE2 protein has a “leucine-zipper” motif that can bind to zein DNA [9], actually recognizing a specific target site on the 22-kD α-zein gene [10]. The mutant gene greatly reduces storage zein protein, thus changing the endosperm texture. The *O2* gene has pleiotropic effects, and its function is complex. Proteomic analysis led Damerval to conclude that the *O2* gene plays a key role in many metabolic pathways [11]. Microarray analysis indicated that 58 genes were up-regulated and 66 genes were down-regulated in *o2* mutants [12]. Transcription profiling using GeneCalling™ indicated that 70 genes corresponding to 23 functional groups were up-regulated and 81 gene fragments belonging to 16 groups were down-regulated in *o2* [13]. Some studies reported differential expression of the *o2* mutant gene in plants with different genetic backgrounds, resulting in varying levels of expressed α-zeins [14] and lysine content [15], [16]. In addition, the OPAQUE2 protein can interact with many other genes or proteins, such as the *cyPPDK* gene [17] and the ribosome-inactivating protein [18].

Pioneer Corporation [19] and the University of Missouri [20] exploited three SSR markers (phi112, umc1066, and phi057) inside the *O2* gene. The three markers can be used to select among the three genotypes, *O2O2*, *O2o2*, and *o2o2*, in maize inbred lines constructed using molecular-assisted selection (MAS).

*Opaque-2* mutants have many poor agronomic characteristics, and QPM was developed to overcome these defects. To increase the lysine content of elite normal maize inbred lines, QPM inbred lines were used as donors to introgress the *o2* mutant gene into elite Chinese normal maize inbred lines using SSR marker phi057. Nearly all of the selected lines presented opaque kernels and elevated lysine concentration. In our previous work, we found that some *o2* near-isogenic lines (NILs) have a wild-type kernel phenotype with unchanged lysine content, such as Liao2345*o2* and Dan598*o2* [21]. Surprisingly, another *o2* NIL derived from liao2345 has an entirely different kernel phenotype with opaque endosperm that is identical to a typical *o2* mutant individuals. In this paper, Liao2345*o2* is referred to as liao2345/*o2*-1, and the second *o2* NIL is referred to as liao2345/*o2*-2.

The *O2* gene is pleiotropic, its function is complex, and some unknown mechanisms of this gene still need to be studied. We were interested in the reason why the two *o2* NILs carry the same CDS but have completely different kernel phenotype. The object of this study was to reveal the mechanisms underlying the reversion of liao2345/*o2*-1.

## Materials and Methods

### Plant materials

Maize inbred line CA339 was used as a non-recurrent parent (donor), and maize inbred line liao2345 was used as a recurrent parent (receptor). CA339 is a QPM inbred line derived from pool33, which is from CIMMYT, and selected by Maize Research Center of Institute of Crop Sciences of CAAS; and liao2345 is a normal elite Chinese inbred line, cultivated by Maize Research Institute of Liaoning Academy of Agricultural Sciences. Two *o2* NILs, liao2345/*o2*-1 and liao2345/*o2*-2, were selected from the two parents by MAS [21]. The pedigree and detailed information refer to Table 1. In each generation, co-dominant SSR marker phi057 was used to select heterozygous (*O2o2*) individuals. The selected heterozygous individuals were self-pollinated and the recessive homozygous (*o2o2*) individuals were selected for further study. SSR marker phi057 was inside the *O2* gene, according to the SSR marker phi057 the genotype of the two isolines was designated as *o2o2*. All of the materials were grown in Beijing during the summer of 2012. All plants were self-pollinated by hand, and the date and time were recorded. The endosperms were picked at approximately noon 18 days after pollination (DAP) and immediately frozen in liquid nitrogen.

**Table 1:** Name and pedigree of maize lines.

Name	Pedigree	Genotype	Endosperm phenotype	Lysine content%
liao2345/*o2*-1	((liao2345×CA339)BC_5_)F_7_	*o2o2*	Hard and vitreous	0.32[21]
liao2345/*o2*-2	((liao2345×CA339)BC_5_)F_7_	*o2o2*	Floury and opaque	0.49
liao2345	Tie7922×Shen5003	*O2O2*	Hard and vitreous	0.28[21]
CA339	pool33	*o2o2*	Semi-hard and semi-vitreous	0.45[44]

### Kernel phenotype and total zein analysis

Seeds were harvested after maturity, and dried in a greenhouse. The kernel appearance and characteristics were recorded using a camera (SONY α700, P mode). Zeins were extracted according to the method described by Wu YR, with slight modification [22]–[25]. For each sample, mature kernels from three ears were mixed evenly, and soaked in distilled water for 6 hours. The embryos and seed coats were removed and the endosperm was finely ground in liquid nitrogen using a mortar. Fifty milligrams of the resultant powder was precisely weighed and transferred to a 2-mL Eppendorf tube and 400 µL of extraction solution that contained 70% ethanol/2% 2-mercaptoethanol (v/v)/28% double distilled water was added to the tube. The mixture was vortexed for 30 s and maintained on a shaking table for at least 6 hours. The mixture was then centrifuged at 13000 rpm (Eppendorf, Centrifuge 5424R) for 15 min and 100 µL of the supernatant was transferred to a new 1.5-mL tube. Next, 10 µL of 10% SDS was added to the supernatant liquid and the liquid was dried in a drying oven at 50°C. One hundred microliters of distilled water was then added in order to resuspend the zeins. Finally, 8-µL samples (equal to 1 mg endosperm powder) were loaded into polyacrylamide gel lanes, separated by a 4% stacking gel and 15% separation gel, and stained by Coomassie brilliant blue R250.

### Chromosome fragment introgression analysis in bin 7.01

In order to analyze the introgression integrity of the *o2* mutant gene, 18 SSR markers were selected in bin 7.01 on the 7th chromosome. All of the markers were selected from the database MaizeGDB (http://www.maizegdb.org/).

DNA samples were extracted from leaves using the improved CTAB method [26]. The quality of the DNA was ascertained by 0.8% agarose gel. The DNA concentration was measured by NANODROP 2000, then diluted to 100 ng/µL. The PCR reaction system contained 200 ng of genomic DNA, 1× Taq Reaction Buffer, 0.2 mM of each dNTP, 0.25 mM of each primer, and 0.025 U/µL of each Taq Polymerase, for a total volume of 20 µL. The touch-down reaction procedure was performed on a C1000™ Thermal Cycler (BIO-RAD). The PCR cycling conditions were as follows: initial denaturation temperature of 94°C for 5 min, followed by 10 cycles of 94°C for 30 s, primer annealing at 65°C for 30 s (every cycle decreased 1°C), and extension at 72°C for 45 s; this was followed by 25 cycles of 94°C for 30 s, primer annealing at 55°C for 30 s, and extension at 72°C for 45 s; and a final extension at 72°C for 10 min. After the PCR procedure, samples were maintained at 4°C until analysis. Electrophoresis and silver staining were performed as described in the CIMMYT laboratory manual protocols [27]. The results were analyzed using the software program GGT [28]. Bands consistent with the recurrent parent origin were recorded as “A,” bands consistent with the non-recurrent parent origin were recorded as “B,” and sites not marked by the markers were recorded as “U.”

### Validation of promoter differences

Several specific primer pairs were designed and used to determine the differences between two *o2* NILs in the promoter region of the *o2* gene. The primers were as follows: oligoF1: 5′-AAAGGTAGTAGAACACACACGGGC-3′, oligoF2: 5′-CGTTGGTAAGGCATGGTGTCTAGCA-3′, oligoF3: 5′-TGCACGTAGTTCGTCGTCCACAT-3′, and oligo R: 5′-CGGTGGTAGCAGCTCCCAGAAGG-3′. All of the primers were designed to amplify the region that included the TATA box of the gene [7]. High-fidelity polymerase (KOD FX DNA Polymerase) was used to amplify the region, and the PCR reaction procedure was as follows: initial denaturation temperature was 94°C for 5 min, followed by 35 cycles of 98°C for 10 s, primer annealing at 55°C for 40 s, extension at 68°C for 80 s; and a final extension at 68°C for 10 min. The PCR products were maintained at 4°C until analysis. The products were separated on a 2.0% agarose gel in TAE buffer.

### Sequence analysis of the promoter and CDS region of the *O2* gene

**Figure 1:**
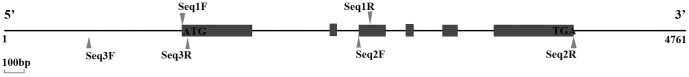
Structure of the *O2* gene and specific site of each sequence primer.

Three pairs of primers that were based on the *O2* gene sequence published in the MaizeSequence database (http://www.maizesequence.org/index.html, GRMZM2G015534) were designed using Primer Premier 5 software. The primers are listed in Table 2 and shown in Figure 1. High-fidelity polymerase (KOD FX DNA Polymerase) was used to amplify the *O2* gene sequence. The cycling conditions were as follows: initial denaturation at 94°C for 5 min, followed by 35 cycles of 98°C for 10 s, primer annealing at 55°C for 45 s, and extension at 68°C for 120 s with a final extension at 68°C for 10 min. The PCR products were maintained at 4°C until analysis. The products were separated on 1.5% agarose gel in TAE buffer. The target genes were then purified and ligated into *pEASY*-Blunt Cloning Vector (Beijing TransGen Biotech Co., Ltd.) using *Escherichia coli* strain DH5α as a host. Plasmids were extracted and sequenced using the commonly used M13F and M13R sequencing primers. The sequences were blasted with the published sequence to ensure that the correct gene was ligated and all the sequences were aligned using DNAMAN version 6 software. And then the sequences were submitted to GenBank, the accession numbers provided by GenBank were KF831423, KF831424, KF831425, and KF831426.

**Table 2:** Primers for target gene fragments.

Names	Amplified Length	Ta Opt °C	Sequences
Seq1F	1373 bp	55	5′ ATGGAGCACGTCATCTCAATGG 3′
Seq1R			5′ TAGTAGCAAGCCTCACGCCAA 3′
Seq2F	1227 bp	57	5′ TAGCAGCATCAGGAATAATCCAGTG 3′
Seq2R			5′ TCAGCGACGCCTGCAAATAA 3′
Seq3F	1419 bp	57.1	5′ TCCTCTTTCCTTCTCTCAGACAGCG 3′
Seq3R			5′ CGGTGGTAGCAGCTCCCAGAAGG 3′

### Analysis of *O2* transcript abundance

The following two pairs of specific primers that spanned the third and the fifth exon of *O2* gene were designed for analysis of transcript abundance: O2RT1-F: 5′-TCAGGAATAATCCAGTGCAGAA-3′, O2RT1-R: 5′-TCGACGTTAGCGTCGTTGTA-3′, O2RT2-F: 5′-TAGCAGCATCAGGAATAATCCAGTG-3′, and O2RT2-R: 5′-CTTAGCTCTTAGGGTCTCCATGTCC-3′. The predicted PCR products were 358 bp and 409 bp. GAPDH gene was selected as the reference gene. The primers used for GAPDH were as follows: GAPDH-F: 5′-CCCTTCATCACCACGGACTAC-3′, and GAPDH-R: 5′-AACCTTCTTGGCACCACCCT-3′. Endosperm collected 18 DAP was ground into fine powder and total RNA was extracted using TRIZol® agent (Invitrogen). An oligo d(T) primer kit was used to reverse transcribe the mRNA (Promega Corporation). A total of 2 µg RNA was used to construct the first strain of cDNA. The reaction system was as follows: 4 µL of 25 mM MgCl_2_, 2 µL of RT 10× Buffer, 2 µL of 10 mM dNTP, 0.5 µL of 2500 U RNAE inhibitor, 0.7 µL of 1500 U AMV RT, and 0.5 µg of oligo d(T) primer, for a total volume of 20 µL. The reaction cycle was 10 min at room temperature, 15 min at 42°C, 5 min at 95°C, and 5 min at 2°C. The reaction mixture was diluted with double distilled water to 50% of its original concentration for PCR detection.

## Results

### Kernel characteristic and total zein analysis

**Figure 2:**
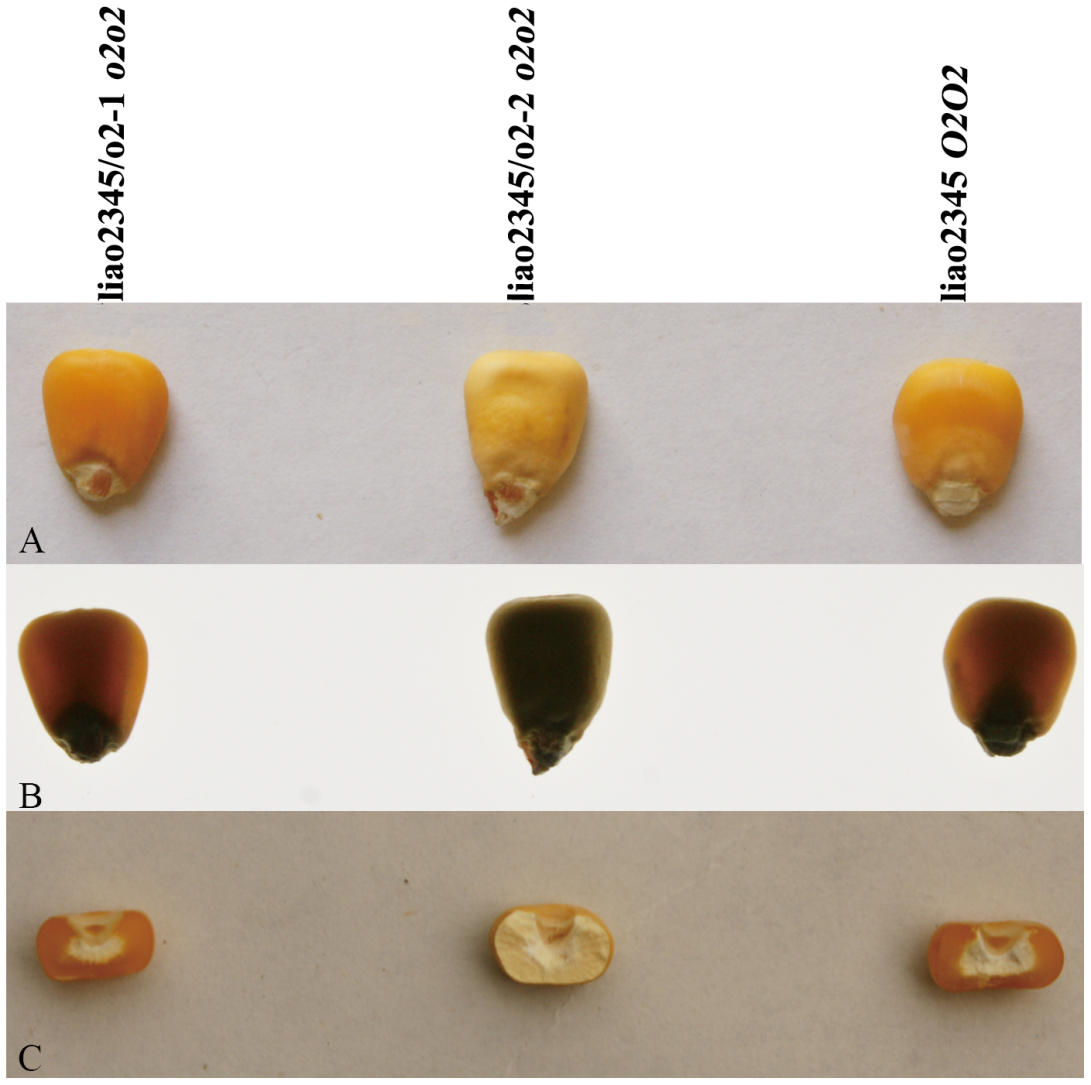
Maize kernel phenotypes of liao2345/*o2*-1, liao23455/*o2*-2, and liao2345; *o2o2*, *o2o2*, *O2O2*. A: Photographs of intact kernels taken under normal light on light box. B: Photographs of intact kernels taken with transmitted light. C: Photographs of decapped kernels taken under normal light.

**Figure 3:**
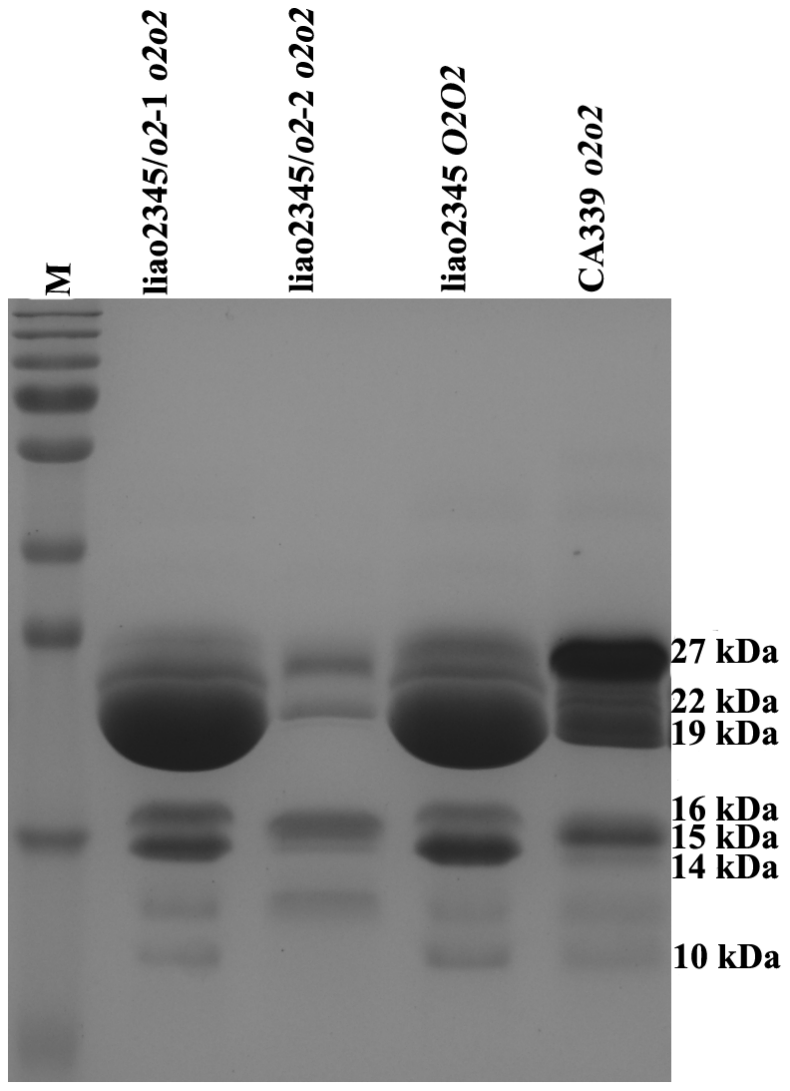
Maize kernel zein accumulation of liao2345/*o2*-1, liao2345/*o2*-2, liao2345, and CA339 in mature seeds, detected by SDS/PAGE. Total zein loaded in each lane is equal to 1 mg of mature seed endosperm, and the relative molecular weights of proteins are marked at the right side, the unit is kDa.

The two *o2* NILs had different kernel phenotypes under normal and transmitted light (Figure 2). The kernels of liao2345/*o2*-1 were hard and vitreous, similar to those of its recurrent parent liao2345, while the kernels of liao2345/*o2*-2 were soft and opaque. Kernel zein concentration and accumulation patterns, as determined by SDS-PAGE, are shown in Figure 3. The relative quantification of zein indicated that the zein concentration of liao2345/*o2*-2 was largely inhibited, while liao2345/*o2*-1 had a zein concentration similar to that of liao2345 and larger than that of CA339. It was clear that the zein accumulation pattern was different among the four lines. The mutant *o2* fragment of CA339 was introgressed into two *o2* NILs. However, the kernel texture and zein accumulation pattern of these lines were completely different from one another. The line liao2345/*o2*-1 had the same pattern as liao2345, whereas liao2345/*o2*-2 had a decreased zein concentration and a different pattern to that of liao2345 and CA339. Compared with liao2345, the *o2* NIL liao2345/*o2*-2 had no 22-kDa α-zein and a very low concentration of 19-kDa α-zein. Both liao2345/*o2*-2 and CA339 had lower concentrations of α-zeins than liao2345 and the α-zeins concentrations of CA339 were higher than that of liao2345/*o2*-2. However CA339 possessed a higher 27-kDa γ-zein concentration than the other three lines.

### Validation of the *o2* mutant gene between the two *o2* NILs and chromosome fragment analysis

**Figure 4:**
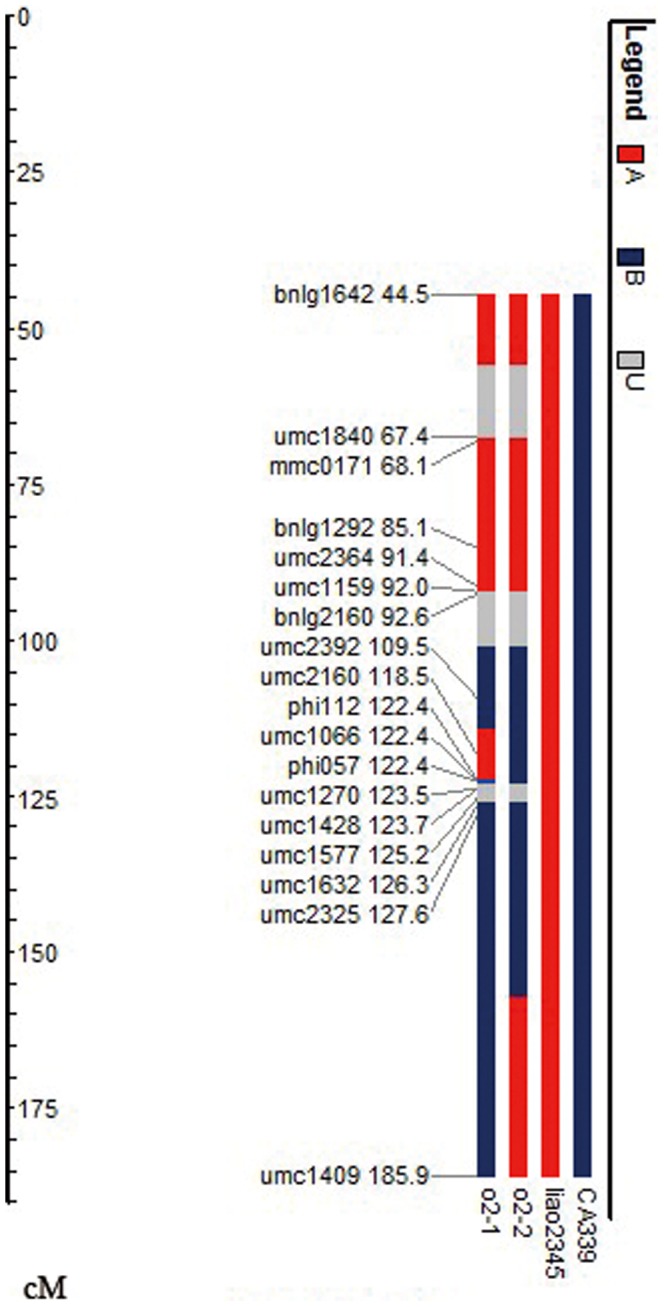
Analysis of chromosome segments introgression of *O2* based on SSR markers mapping to bin7.01. The positions of SSR markers are based on a map from IBM2 2008 Neighbors 7. The red fragments (A) are of recurrent parent origin, blue fragments (B) are of donor origin, and gray fragments (U) represent markers that have no polymorphism between the two parents, and have no information for that genotype.

The SSR marker phi057 indicated that the two *o2* NILs possessed the *o2* mutant gene; however, they displayed completely different kernel phenotypes. We analyzed the chromosome segments in bin7.01 (Figure 4). Eighteen SSR markers were selected for examination of the two *o2* NILs. Of these markers, five of them had no polymorphism or could not mark the corresponding genotype; five indicated that both NILs had segments from the recurrent parent (Figure 4, red region); five indicated that both *o2* NILs had segments from the non-recurrent parent (Figure 4, blue region); and three markers, umc2160, phi112, and umc1409, displayed polymorphisms between the two *o2* NILs. The line liao2345/*o2*-1 had fragments from the recurrent parent, liao2345, at the site of umc2160 and phi112, while liao2345/*o2*-2 had fragments from the non-recurrent parent, CA339, at these two sites. At the site of umc1409, liao2345/*o2*-1 had fragments from CA339 and liao2345/*o2*-2 had fragments from liao2345.

**Figure 5:**
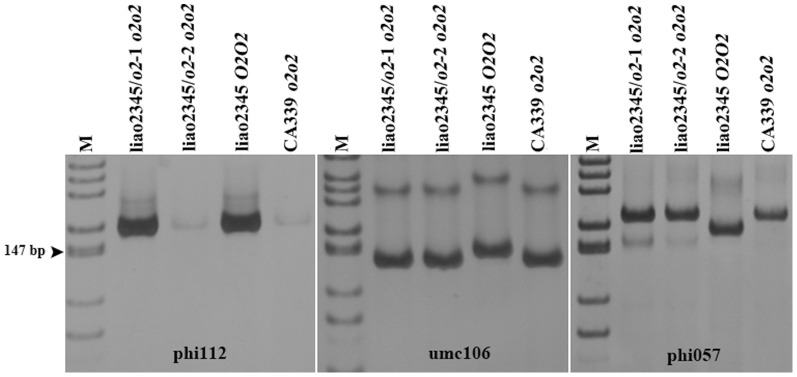
Test of three SSR markers inside the *O2* gene by PAGE. phi112, umc1066, and phi057 can distinguish the three genotype of the *O2* gene. The amplified region of phi112 located in the promoter of *O2* the gene; and umc1066 in the first exon of the *O2* gene; and phi057 in the sixth exon of the *O2* gene.

Three markers at position 122.4 were used: phi057 was a co-dominant marker that was located in the sixth exon, and was used to select the *o2* NILs in this study; umc1066 was also a co-dominant marker, located in the first exon, but was not widely used for MAS because no polymorphisms were found among many materials; and phi112 was located near the promoter. The results demonstrated that the *o2* gene of liao2345/*o2*-1 and liao2345/*o2*-2 were the same between the first exon and the sixth exon (Figure 5, umc1066 and phi057). However, liao2345/*o2*-1 and liao2345/*o2*-2 displayed differences at the promoter region (Figure 5, phi112): liao2345/*o2*-1 had the promoter of its recurrent parent liao2345, whereas liao2345/*o2*-2 had the promoter of its non-recurrent parent, CA339. We concluded that the two *o2* NILs have different promoters.

### Validation of promoter differences

**Figure 6:**
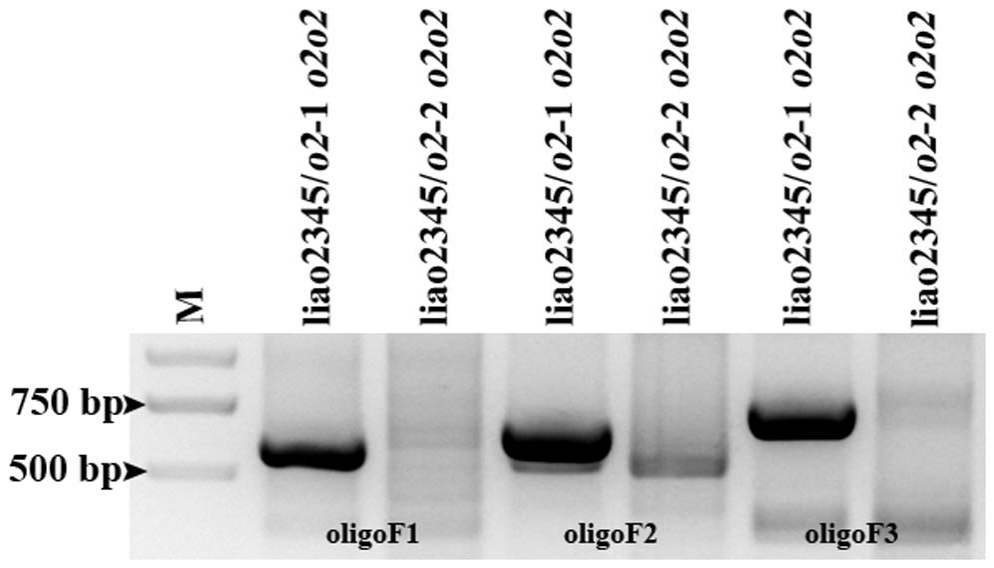
Analysis of promoter difference between two *o2* NILs by normal PCR on agarose gel. The size of the amplified region of three pairs primers are 582 bp, 648 bp, and 723 bp. This region contains the TATA box and initiation codon of *O2* gene.

To verify that liao2345/*o2*-1 and liao2345/*o2*-2 had differences in the promoter region, several primers were used to examine the two *o2* NILs. oligo F1, oligo F2, oligo F3, and oligo R were designed to form three pairs of primers that were used to amplify the promoter region of liao2345/*o2*-1 and liao2345/*o2*-2. The target DNA fragments of liao2345/*o2*-1 were amplified, while those of liao2345/*o2*-2 were not (Figure 6), confirming that liao2345/*o2*-1 and liao2345/*o2*-2 contained different promoters.

### Sequence analysis

**Figure 7:**
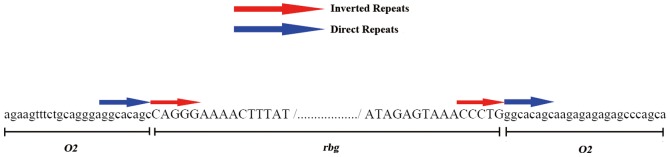
The 5′ flanking sequence of the *o2* gene of liao2345/o2-2. The capital letters represent the sequences of the *rbg* transposon, and the lower case letters represent the flanking sequences of the *O2* gene. Red arrows indicate two inverted repeats of the *rbg* transposable element, and the blue arrows indicate two direct repeats, one of which was created by the insertion of *rbg*.

The primer pairs Seq3F and Seq3R also did not amplify the promoter regions of liao2345/*o2*-2 and CA339. However, they could be used to amplify the promoter regions of liao2345/*o2*-1 and liao2345. Moreover, the products were sequenced and aligned with the B73 sequence and the identity was 100%. Asymmetric interlaced PCR was used to determine the 5′ flanking sequence of the liao2345/*o2*-2 and CA339 promoters. The mutant sequence was blasted in NCBI and had high homology with the *Bg* transposable element. It is the *rbg* transposable element that is inserted in the locus [38]–[41]. The 5′ and 3′ sequence of *rbg* and the *O2* flanking sequence of liao2345/*o2*-2 and CA339 were cloned (Figure 7), and we verified that the *rbg* transposable element was inserted into the promoter regions of the *o2* gene of liao2345/*o2*-2 and CA339. And the partial sequence of *rbg* was submitted to GenBank, the accession number is KF831427. The insertion created an 8-bp direct repeat (GGCACAGC), and *rbg* had a 5-bp inverted repeat. The insertion was located between the TATA box and initiator codon, and was exactly 250 bp from the initiator codon (ATG).

**Figure 8:**
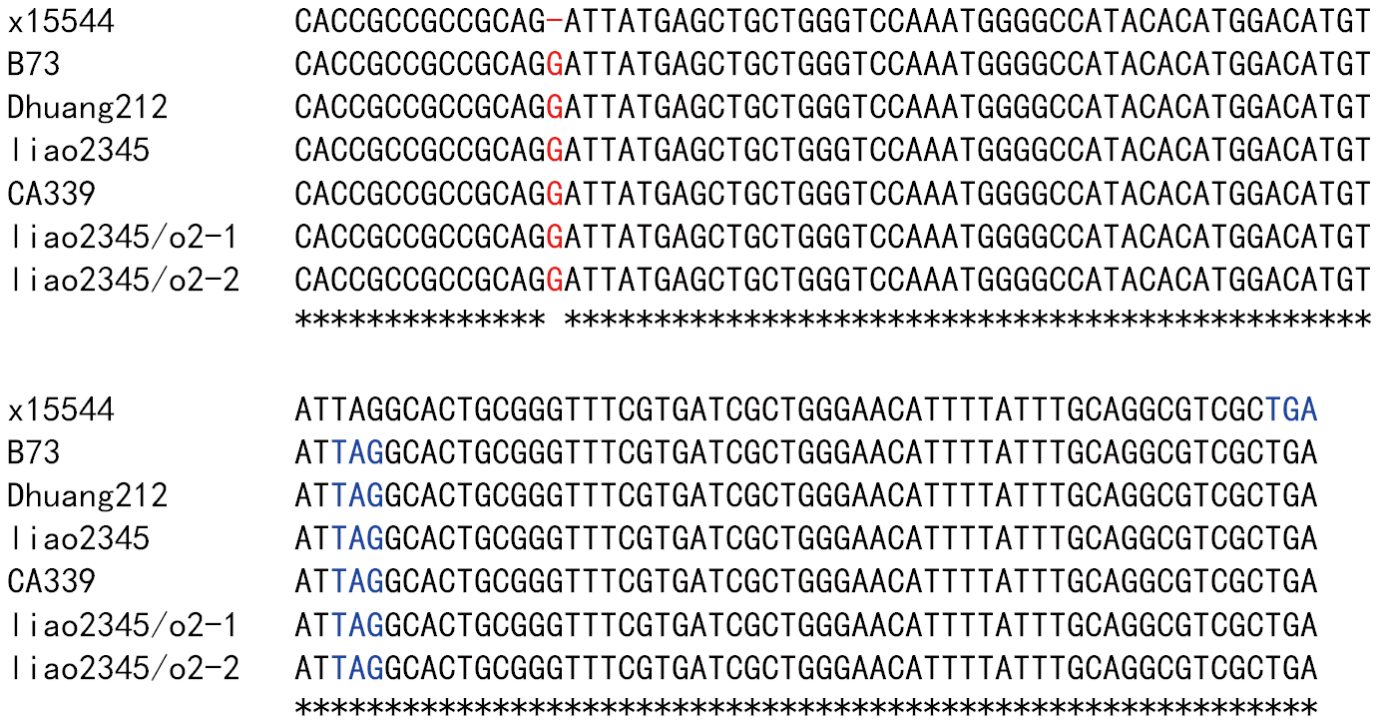
Multiple alignment of partial sequences of the sixth exon of the *O2* gene. Sequences of x15544 and B73 are supplied by GenBank and MaizeSequence, respectively. The other five sequences are sequenced by us. Dhuang212 and liao2345 are wild-type inbred lines in China. Dashes indicate gaps and asterisks indicate identical residues. The stop codons are indicated by blue characters, and the insertions and deletions are indicated by red characters.

The sequences of the *O2* alleles, from the initiator codon (ATG) to the stop codon (TAG), were cloned and sequenced, including six exons and five introns. Figure 8 displayed multiple alignment of the 117 bp of the sixth exon. This alignment indicated that x15544 (supplied by GenBank) has -1 frameshift compare to the other six sequences. However, B73, Dhuang212, and liao2345 are wild-type inbred lines. Moreover, the B73 sequences were supplied by the database MaizeSequence (GRMZM2G015534) [29]; therefore the stop codon should be a TAG, not a TGA.

**Figure 9:**
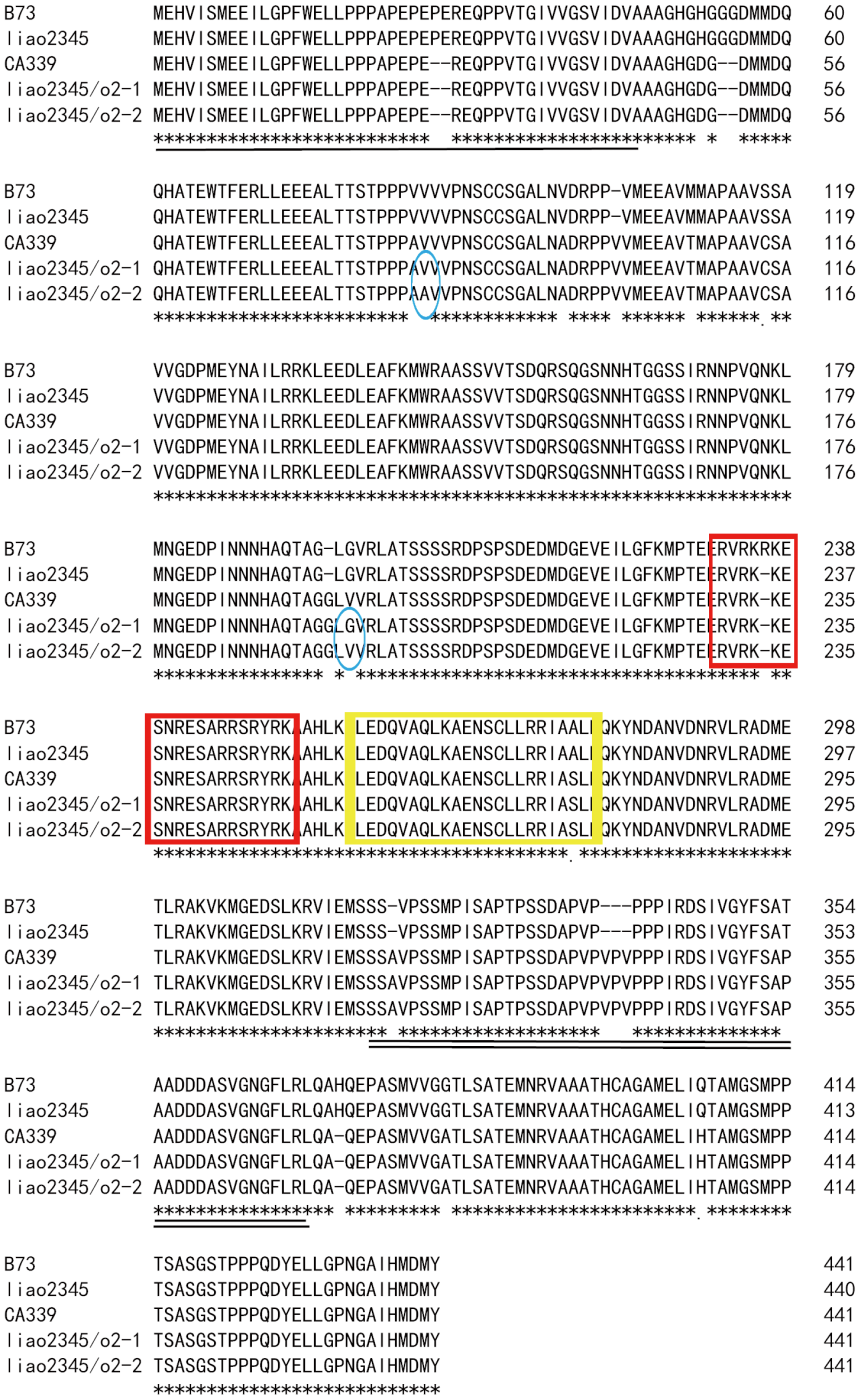
Multiple alignment of deduced OPAQUE2 protein sequences. Dashes indicate gaps and asterisks indicate identical residues. The SSR site of umc1066 is underlined and the SSR site of phi057 is indicated by double underlining. The basic motif is marked with a red box, and the leucine zipper domain is marked by a yellow box. Blue ellipses indicate sites that differ between the two *o2* NILs.

Using the published sequence of B73, a wild-type inbred line, we deduced the OPAQUE2 protein sequence. Multiple alignment analysis (Figure 9) indicated that both of the *o2* NILs acquired the donor’s (CA339) coding sequence and were different from their recurrent parent (liao2345). In comparison to liao2345, liao2345/*o2*-1 and liao2345/*o2*-2 had five amino acid deletions, six amino acids insertions, and 10 amino acid substitutions. Interestingly, there were only two sites at which amino acids substitutions occurred between the two *o2* NILs. At these sites, a valine (V) was substituted by an alanine (A) and a glycine (G) was substituted by a valine (V). All of these amino acids are non-polar and hydrophobic; consequently, they may not affect the structure of the OPAQUE2 protein. Further, in the OPAQUE2 protein, the leucine zipper domain of the two *o2* NILs includes a substitution of a non-polar hydrophobic alanine (A) to a polar hydrophilic serine (S). The identity between B73 and liao2345 is 99.77%, whereas the identity between CA339 and liao2345 is 96.27%. Clearly, there are differences between the mutant gene and the wild-type gene, but the differences are not large and do not affect the function of OPAQUE2 protein.

### Analysis of *O2* transcript abundance

**Figure 10:**
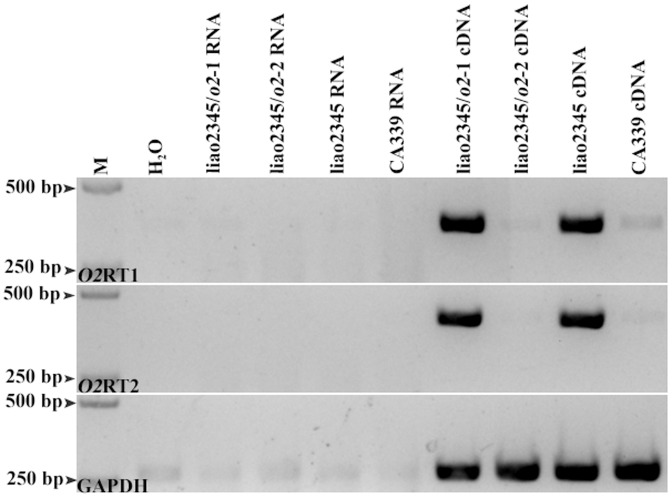
Examination of the *O2* gene transcript abundance in developing endosperm (18 DAP) by RT-PCR. O2RT1 and O2RT2 are two specific primers spanning the third and the fifth exon of the *O2* gene, the amplified region of ORT2 is larger than that of O2RT1; GAPDH is the reference gene. The templates of RT-PCR are labeled in each line, H_2_O represents the blank control; RNA represents the negative control; cDNA is used to detect abundance of the *O2* transcript.

Two pairs of specific primers were constructed to examine the transcript abundance of the *O2* gene: both of them spanned the third and the fifth exon of the *O2* gene. The transcript quantity of the *O2* gene was obviously different between the two *o2* NILs: liao2345, liao2345/*o2*-1 had the same level of transcript, whereas the transcript of liao2345/*o2*-2 and CA339 were largely inhibited (Figure 10). The results demonstrated that the insertion of the *rbg* transposable element inhibited the transcription of the *O2* gene. The transposon knocked down the expression of the *O2* gene, and this was sufficient to induce a mutant phenotype.

## Discussion

Detection of SSR marker phi057 indicated that two *o2* NILs had successfully obtained the *o2* mutant gene of CA339, so the two *o2* NILs were designated as *o2* mutant lines, and their genotype was *o2o2*. The germplasm of QPMs are associated with a recessive *o2* mutant gene and modifier genes (*o2* modifiers) [30], [31]. These genes are responsible for the production of high lysine content and hard vitreous or semi-vitreous endosperm. The *o2* modifiers can increase γ-zein content by 2- to 3-fold [32], [33] and the mechanism of this increase has been studied using RNAi [22], [34], [35]. In our study, the kernel phenotype (Figure 2) and the zein accumulation pattern (Figure 3) indicated that the 19- and 22-kDa α-zeins were inhibited in liao2345/*o2*-2 and the 22-kDa α-zein was almost undetectable, resulting in kernels that were opaque and soft. We speculated that the background of liao2345 contained no *o2* modifiers. Therefore, the maize inbred line, liao2345/*o2*-2, is not a QPM inbred line. The maize inbred line liao2345/*o2*-1 is also not a QPM inbred line because its lysine and γ-zein concentrations were lower than those of CA339. In our previous study, liao2345/*o2*-1 was determined to be *o2* non-high lysine maize [21].

Chromosome fragment analysis demonstrated that the two *o2* NILs had different exchange patterns in bin 7.01. SSR markers umc2160 and phi112 indicated that liao2345/*o2*-1 had a large chromosome fragment of liao2345 (Figure 4). Recombination occurs during introgressing and backcrossing and progeny will have different recombination patterns. A very low frequency of recombination occurred inside the *O2* gene, which means that the *o2* gene of liao2345/*o2*-1 was a fused gene, with a promoter derived from liao2345 and CDS derived from CA339. However, liao2345/*o2*-2 inherited the whole mutant *o2* gene from CA339. The results indicate that this may be the reason that two *o2* NILs have different kernel phenotypes.

In contrast to the published sequence (GenBank Accession No. X15544), our previous study indicated that the OPAQUE2 protein of CA339 consisted of only 442 amino acid residues, shorter than that of X15544, due to a frameshift, resulting in the truncation of the coding sequence [21]. However, the newly published sequence of the B73 inbred line shows that there is no frame-shift mutation in CA339. In three wild-type inbred lines, B73, Dhuang212, and liao2345, the real stop codon is TAG instead of TGA (Figure 8). We retrieved another 47 partial sequences of the maize *o2* gene from GenBank. Only two of these sequences were the same as x15544 and the three partial sequences had -1 frameshift relative to the other sequences. The shifted partial sequences are NM 001111951.1, x15544, and AY109364. We found that two *o2* NILs, liao2345/*o2*-1 and liao2345/*o2*-2, contained the same coding sequence of CA339. Between these two *o2* NILs, there were two amino acid substitutions, valine (V) to alanine (A) and glycine (G) to valine (V). In both cases, the amino acids were non-polar and hydrophobic. Moreover, these amino acids are not in the basic motif of the OPAQUE2 protein and may not affect the function of the OPAQUE2 protein. Further study is needed to identify whether these changes disrupt the function of the OPAQUE2 protein. A single amino acid substitution mutation from a non-polar hydrophobic alanine (A) to a polar hydrophilic serine (S) was found in the leucine zipper domain. This mutation might cause the OPAQUE2 protein to lose its spatial conformation, and thus, lose its function of transcriptional activation. However, there was no evidence as to whether this mutation might cause a loss of function in the OPAQUE2 protein.

Transposable elements were first reported by McClintock [36]–[38]. Many transposable element systems have been characterized in maize. Transposable elements have many genetic effects, including mutation, chromosomal deletions, and rearrangements. The *Bg-rbg* transposable elements system is responsible for the instability of the mutable allele *o2m(r)* at the *O2* locus [39], [40]. *Bg* is an autonomous element and *rbg* is a non-autonomous element that is specific to the *O2* gene. Nine *o2* alleles were detected in the presence of an active autonomous *Bg* transposable element and five of these responded to the presence of *Bg* [41]. This phenomenon indicates that some *o2* mutants were formed by the insertion of *rbg* transposable element. This type of mutation can be classified into a null mutation. In our study, we determined that liao2345/*o2*-2 and CA339 contained the *rbg* transposable element (Figure 7). The *rbg* has been cloned and sequenced [42]. It contains 4500 nucleotide base pairs, making it easy to identify. Such a large fragment inserted in the promoter of the *O2* gene could affect the transcript of the *O2* gene, and we suspected that this insertion greatly inhibits the transcript abundance of the *O2* gene. Our suspicion was then verified, as shown in Figure 10, the *O2* gene of liao2345/*o2*-1 and liao2345 were normally transcribed, while the transcript of liao2345/*o2*-2 and CA339 were inhibited to a large degree. We concluded that the two *o2* NILs have the same coding sequence of CA339, and the two *o2* NILs have different kernel phenotypes because they have different promoters: liao2345/*o2*-1 has the wild-type promoter, while liao2345/*o2*-2 has the mutant promoter, as determined by their different patterns of recombination. We deduced that the OPAQUE2 protein of CA339 had the normal function of maintaining vitreous and hard endosperm. However, further studies, such as proteomic analyses, are required to determine whether the mutant OPAQUE2 protein has the same function as the wild-type OPAQUE2 protein. The unique maize inbred lines, liao2345/*o2*-1, liao2345/*o2*-2, and liao2345 can help us to understand the role of the *O2* gene, and its regulatory network in maize.

We surmise that the reversion of liao2345/*o2*-1 to wild type was due to the recombination with the wild-type liao2345 promoter during introgression and backcrossing. Because the promoter sequences of liao2345 and liao2345/*o2*-1 are identical. The promoter sequences may be different between the two lines, if it was due to the excision of the *rbg* element. The insertion of *rbg* in *O2* locus will create a 8-bp direct repeat, and the inserted base pairs may not be excised with the *rbg* element. Moreover, *rbg* is a nonautonomous element, and excision will not occur when the *Bg* element is not present in the background. The liao2345/*o2*-2 is very stable and did not revert; therefore, it contains no active *Bg* element. Only when reversion occurs prior to the first mitotic division of the primary triploid endosperm nucleus, will whole endosperm revertants (WER) be formed. Therefore, it is recombination that forms the reversion, not the excision of *rbg*. For the instability at the *o2* locus [39], we need to use SSR marker phi112 when introgressing the *o2* mutant gene into normal inbred lines to ensure that we have introgressed the mutant promoter. However, phi112 is a dominant marker, and can not be used to distinguish homozygotes from heterozygotes. The results of the present study indicate that SSR marker phi057 is not a functional marker for the *o2* gene. Even though there is polymorphism between receptors and donors in the simple sequence repeat region of phi057, the function of the *O2* gene is not determined by this locus. Some wild-type maize lines, Duohuang29, Dan3130, and 9046, have no polymorphisms with CA339 [43], which indicates that they have the same sequence as CA339 at this locus. This further demonstrates that sequence variations at this site did not affect the function of OPAQUE2 protein. Our previous study demonstrated that there are two types of *o2* mutant individuals: inbred lines CA339 and CA335 are different from Shandong2548 and Qi205, and have two different types of *o2* mutant gene [44]. Functional markers should therefore be exploited according to specific *o2* mutant genes, particularly SSR and CAPS markers, for MAS breeding of QPM.
